# Use of Lateral Calcaneal Flap for Coverage of Hindfoot Defects: An Anatomical Appraisal

**DOI:** 10.1155/2015/212757

**Published:** 2015-11-11

**Authors:** Panagiotis Zygouris, Adamantios Michalinos, Vassilis Protogerou, Evangelos Kotsiomitis, Antonios Mazarakis, Ioannis Dimovelis, Theodore Troupis

**Affiliations:** Department of Anatomy, National and Kapodistrian University of Athens, Mikras Asias 75 Street, Goudi, 11527 Athens, Greece

## Abstract

Lateral calcaneal flap is an established surgical option for coverage of lateral calcaneum and posterior heel defects. Lateral calcaneal flap vascularization and innervations are based on lateral calcaneal artery neurovascular bundle, that is, lateral calcaneal artery, small saphenous vein, and sural nerve. Anatomical research has allowed exploration of its many advantages but can also lead to its various modifications, permitting a wide variety of clinical applications. In this paper the authors report an anatomical and clinical study on lateral calcaneal artery course and lateral calcaneal flap clinical applications. Anatomic part of our study focused on lateral calcaneal artery course and optimization of surgical technique for flap harvesting. Data were used for design of lateral calcaneal flap in 5 patients. Our results were satisfactory in terms of coverage adequacy, perioperative morbidity, and functional and aesthetical outcome.

## 1. Introduction

Posterior heel and lateral calcaneum defects are often difficult in their restoration because of their osseous or tendinous bed, poor area vascularization, continuous movement, and high functional demands. Conservative treatment usually fails; use of split of full-thickness skin grafts often leads to unacceptable results while free flaps transfer is technically demanding and presents significant perioperative morbidity [1, 2].

Lateral calcaneal flap is based on lateral calcaneal artery (LCA) neurovascular bundle consisting of LCA, small saphenous vein, and sural nerve. Lateral calcaneal flap is an important surgical option for coverage of hindfoot defects. Its advantages include adequate coverage of posterior heel and lateral calcaneum [3], high success rate, low perioperative morbidity [4], and good functional results [5]. Investigation of LCA anatomy has led to various modifications of lateral calcaneal flap, including its island form [6], its adipofascial form [7], its reverse form [8], and its V-Y advancement form [9].

While being widely used, anatomical details on lateral calcaneal flap vascularization are still lacking, yet their impact is important for final result. The aim of this study is to investigate LCA anatomy and lateral calcaneal flap clinical results.

## 2. Materials and Methods

Twelve cadaveric legs were dissected for creation of lateral calcaneal flap at the Laboratory of Forensic Medicine and Toxicology, National and Kapodistrian University of Athens, Greece. Flap was dissected at its short straight form at 6 legs and at its long curved at 6 legs. We based the design of the flap on the estimated course of the LCA, as described in morphometric studies [4, 10], using 3 anatomical landmarks. First landmark was origin of LCA from peroneal artery, approximately 6 cm above the middle of the line connecting lateral malleolus and insertion of Achilles tendon. Second landmark was the middle of the line connecting between lateral malleolus and insertion of the Achilles tendon. Third landmark was 1 cm proximal to the tuberosity of the fifth metatarsal. Based on those 3 landmarks, curved course of LCA was designed and flap, in its short straight or long curved form, was designed. Dissection of the flap began from distal to proximal. As soon as LCA was identified, it was ligated and injected with methylene blue. Then dissection continued along the course of LCA from distal to proximal so as to identify its area of vascularization. Distance between lateral malleolus and LCA was measured, as was flap's ideal pivot point.

**Figure 1:**
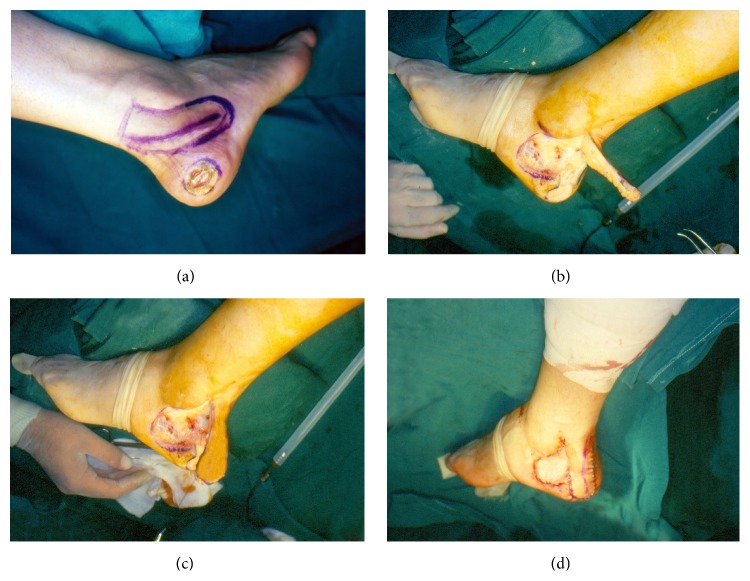
Lateral calcaneum defect covered by lateral calcaneal flap, straight form. (a) Preoperative design of lateral calcaneal flap. (b) Harvesting of the flap. (c) Alignment of the flap. (d) Immediate postoperative result.

**Figure 2:**
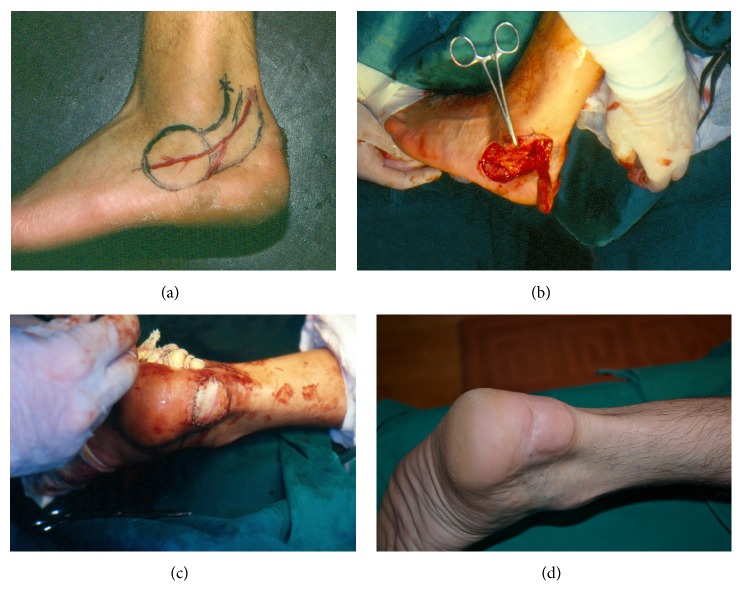
Posterior heel defect covered by lateral calcaneal flap, island form. (a) Preoperative design of the flap. (b) Harvesting of the flap. (c) Immediate postoperative result. (d) Long-term result.

Clinical part of the study included use of lateral calcaneal flap for rerepair of defects of posterior heel in 5 patients between 2005 and 2010. Mean age of the patients was 33 years old (20–62). All defects were consequence of motorcycle injury. In all patients defect had been covered in the past with split thickness skin grafts. Result was unacceptable in terms of functionality and appearance because of hyperkeratosis (3 patients) and ulceration (2 patients). One of our patients suffered from moderate atherosclerosis. Apart from that, no other health issues were encountered (Figures 1 and 2).

Preoperatively course of LCA was outlined with Doppler ultrasound. Surgical procedure was performed under a tourniquet and surgical loupes of 4.3 magnification.

The defect was measured at its two maximal dimensions and then the flap was marked on the skin (as a transposition or island flap). The damaged skin graft was totally removed; the defect was measured and the required size of the flap was marked on the skin as a skin transposition or island flap. When a skin transposition flap was used an incision was made to the skin until under the fascia. The incision at the posterior edge of the flap was carried down to the periosteum close to the Achilles tendon and near the calcaneum under the inferior border of the line connecting lateral malleolus and Achilles tendon. Undermining of the fascia was straightforward when the dissection was done immediately over the periosteum. The flap was raised proximally to the pivot point and was rotated to cover the defect.

When an island flap was used the surgical procedure to elevate the fascial side of the flap was similar to its long version. The differences were the carrying skin, the pedicle preparation, and the tunnel we created so that the flap could approach the defect. For the pedicle construction an incision was made posterior to the lateral malleolus and extended proximally along the fibula and the peroneus longus and the pedicle was separated from the overlying skin. The sural nerve and the accompanying vessels in the suprafascial layer were identified and kept into the pedicle of the flap and a tunnel was created to approach the defect.

Middle size of the defects was 4 × 4.5 cm. Donor site was covered with full-thickness skin graft.

## 3. Results

**Figure 3:**
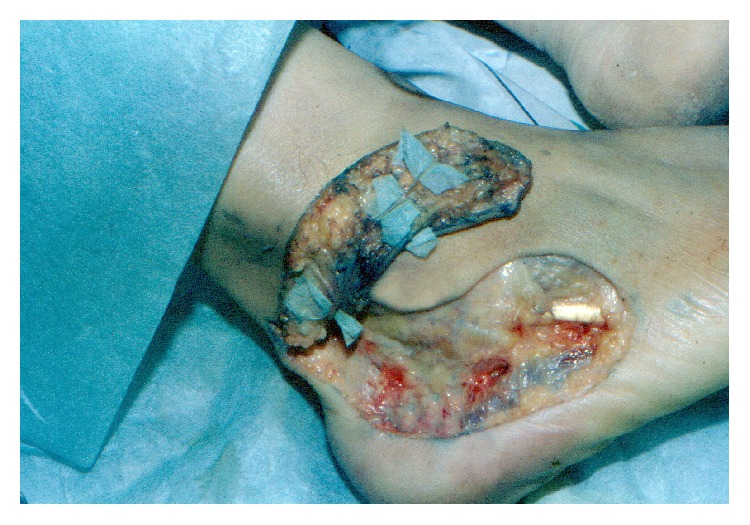
Cadaveric dissection of lateral calcaneal flap, showing LCA (left) and small saphenous vein (SSV) (right).

LCA was found 1.1 cm behind lateral malleolus, on the line connecting lateral malleolus with insertion of Achilles tendon, above deep fascia. Small saphenous vein coursed subcutaneously, parallel with sural nerve and above LCA. Based on visualized branches of LCA, ideal size of the flap was measured at 7-8 cm length for its straight form and 12-13 cm length for its curved form. Width was measured at 4 cm and pivot point at 3 cm above the inferior border of lateral malleolus. Size and dimensions of lateral calcaneal flap are adequate for coverage of deficits of posterior heel (Figure 3).

Good correspondence was always found between estimated course of LCA and its course radiographically and surgically identified. Based on that, dissection should always start outside the triangle bounded by inferior border of lateral calcaneus, Achilles tendon, and a point about 6 cm above the line connecting those two points. Dissection should proceed to the level of the deep fascia early since depth of LCA course and its branches is not stable. A generous lateral margin should be left since the donor area can usually be covered satisfactorily and the flap can be trimmed before its final positioning.

Concerning surgical results, only the atherosclerotic patients presented venous edema and a small area of superficial necrosis, treated conservatively. Apart from that, no morbidity concerning the flap and the donor region appeared. All operated patients were ambulatory at 8th postoperative day. Sensitivity of the flap was conserved intact except for 2 points discrimination in the lateral part of the dorsum of the foot. All patients are able to wear shoes and no restriction concerning leg movement has been observed. Follow-up of the patients ranges between 5 months and 10 years (Table 1).

## 4. Discussion

Reconstruction of soft tissue defects of calcaneal region and posterior heel is demanding because of their osseous or tendinous bed, poor vascularization, and constant area movement. Their restoration necessitates tissues with reliable blood supply, adequate elasticity, and sensitivity. Furthermore they are often of traumatic origin and endorse young persons, causing significant morbidity if suboptimally treated.

Lateral calcaneal flap was introduced by Grabb and Argenta in 1981 [11]. Based on course and distribution of LCA they described two possible forms, short straight and long curved. While being widely used, relative few anatomical details are known over flap's vascularization.

Peroneal artery divides approximately 6 cm above the tip of the lateral malleolus into LCA and its anterior perforating branch [5]. LCA courses downward and penetrates deep fascia at a distance of 3.8 cm (range: 3–4.5 cm) above the tip of lateral malleolus [3]. It acquires a deeper yet almost parallel course to small saphenous vein and sural nerve, forming the neurovascular bundle of lateral calcaneal flap. Small saphenous vein and sural nerve course at the level of the superficial fascia [3]. At the level of the ankle, LCA follows a curve lying approximately in the middle of the distance between tip of lateral malleolus and insertion of Achilles tendon. Through this part various branches follow a downward and posterior course and form a superficial course around calcaneum. LCA continues distally until the tip of the fifth metatarsal and there it divides into its dorsal branch that anastomoses with lateral tarsal artery and its plantar branch that anastomoses with lateral plantar artery [2, 8]. In 12% of the cases LCA is a branch of posterior tibial artery [4].

From a surgical point of view, most important morphometric characteristics of LCA are its depth from skin level, its adequacy as arterial network, and its distance from surgically important landmarks like the tip of the lateral malleolus. Distance between LCA and tip of lateral malleolus was calculated at 1 cm by Demirseren et al. [2]. The same distance was measured at 3 cm according to Freeman et al. [4] and at 2.6 cm according to Elsaidy and El-Shafey [3]. Distance of LCA from Achilles tendon was measured at 1.35 cm from Borrelli and Lashgari [10] and at 5–8 mm from Demirseren et al. [2]. Diameter of LCA is measured at 1.75 ± 0.12 mm and its depth at 7.5–8 cm [3]. Distance between LCA and sural nerve has been measured at approximately 1 cm [8, 12]. Based on the above measurements, Elsaidy and El-Shafey described a “danger triangle” during dissection of LCA between Achilles tendon, lateral malleolus, and penetration point of deep fascia from LCA [3].

Based on the anatomic characteristics of the LCA, various modifications of the lateral calcaneal flap have been invented. In 1984 Holmes and Rayner [6] utilizing the length and stable caliper of LCA introduced the island form of lateral calcaneal flap. Its longer pedicle allows coverage of defects of anterior malleolus, posterior aspect of calcaneum, and Achilles tendon. Island form prevents problems like kinking of the pedicle, dog-ear deformity, and the need for sacrificing the normal skin bridge for flap insetting. Undermining of the skin during flap creation might lead to necrosis [2]. Ishikawa et al. [8] studied the rich anastomotic network of LCA with lateral plantar artery and lateral tarsal artery and invented the reverse lateral calcaneal flap. Reverse lateral calcaneal flap's pivot point is positioned about 20 mm proximal to fifth metatarsal and its proximal edge one or two fingers proximal to inferior portion of lateral malleolus. Its vascularization does not depend on peroneal artery and thus should be meticulously tested for adequacy both preoperatively with Doppler ultrasound and intraoperatively with vascular clamping of LCA. Its diminished vascularization makes it inappropriate for atherosclerotic patients. Furthermore mandatory division of sural nerve compromises flap's sensitivity. Lin et al. in 1996 [7] designed the adipose lateral calcaneal flap utilizing the deep position of LCA. Adipose lateral calcaneal flap's softness and pliability provides a beneficial filling for defects after debridement and minimizes donor size morbidity. Its small pedicle and size make it improper for coverage of large defects [13].

## 5. Conclusions

Lateral calcaneal flap presents good functional and aesthetical results for coverage of posterior heel and lateral calcaneum defects. Its advantages are its good functional results, its small perioperative morbidity, and its relative easiness for its design and creation. It is also a flap resistant to atherosclerosis, a common problem in patients with posterior heel defects.

Good knowledge of LCA anatomy ensures optimal design of the flap and maximum utilization of its length, protects from operative accidents including accidental devascularization or denervation of the flap, and allows optimum usage of lateral calcaneal flap various form, in respect to defect's particular characteristics.

Parallel anatomical and surgical research allows surgeon to benefit from classical anatomical knowledge, put it in use, and ensure the best for their patients' interests.

## Figures and Tables

**Table 1:** 

Number	Sex, age	Defect	Previous operation	Comorbidities	Flap form	Follow-up (y)	Complications
1	M, 20	Posterior heel, Achilles tendon—ulceration	Split thickness graft	No	Island	1	None

2	M, 30	Achilles tendon—hyperkeratosis	Split thickness graft	No	Short straight	0.5	None

3	M, 27	Posterior heel, Achilles tendon—hyperkeratosis	Split thickness graft	No	Long straight	1	None

4	M, 26	Posterior heel, Achilles tendon—ulceration	Split thickness graft	No	Island	10	None

5	M, 62	Posterior heel defect—ulceration, hyperkeratosis	Split thickness graft	Atherosclerosis	Long straight	3	Venous oedema—superficial necrosis
